# A Detailed Protocol for the Induction of Anemia and RBC Transfusion–associated Necrotizing Enterocolitis in Neonatal Mice

**DOI:** 10.21769/BioProtoc.4993

**Published:** 2024-05-20

**Authors:** Balamurugan Ramatchandirin, Marie Amalie Balamurugan, Suneetha Desiraju, Yerin Chung, Krishnan MohanKumar

**Affiliations:** 1 Department of Biochemistry and Molecular Biology, University of Nebraska Medical Center, Omaha, NE, USA; 2 Department of Pediatrics, Johns Hopkins University School of Medicine, Baltimore, MD, USA; 3 Childrens Health and Research Institute, University of Nebraska Medical Center, Omaha, NE, USA; 4 Department of Pediatrics, University of Nebraska Medical Center, Omaha, NE, USA

**Keywords:** Phlebotomy-induced anemia, RBC transfusion, Necrotizing enterocolitis, Neonatal mice, Intestinal injury

## Abstract

Anemia is a common and serious health problem, nearly universally diagnosed in preterm infants, and is associated with increased morbidity and mortality worldwide. Red blood cell (RBC) transfusion is a lifesaving and mainstay therapy; however, it has critical adverse effects. One consequence is necrotizing enterocolitis (NEC), an inflammatory bowel necrosis disease in preterm infants. The murine model of phlebotomy-induced anemia and RBC transfusion–associated NEC enables a detailed study of the molecular mechanisms underlying these morbidities and the evaluation of potential new therapeutic strategies. This protocol describes a detailed procedure for obtaining murine pups with phlebotomy-induced anemia and delivering an RBC transfusion that develops NEC.

**Graphical overview**


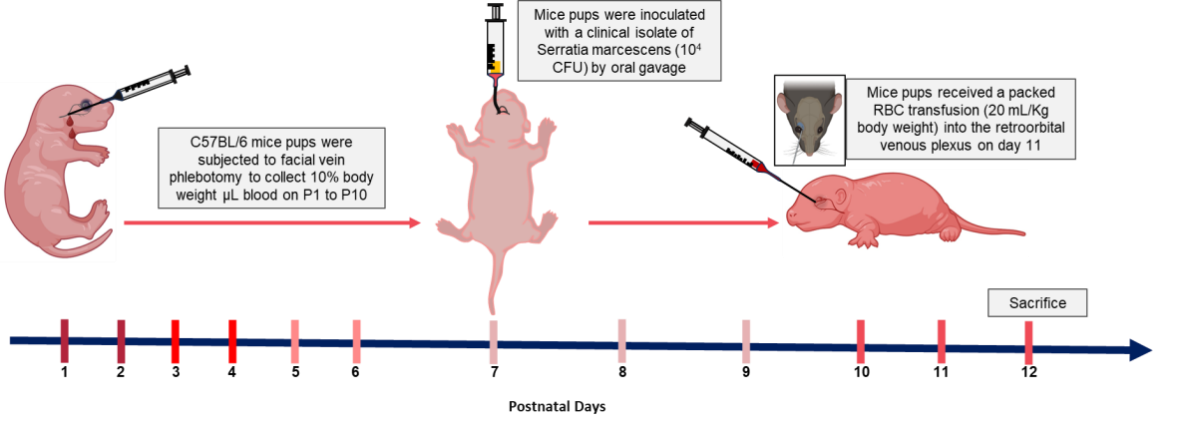


**Schematic diagram of murine model of anemic and red blood cell (RBC) transfusion-associated necrotizing enterocolitis (NEC)**

## Background

Red blood cell (RBC) transfusions are necessary and essential therapeutic interventions frequently utilized in the neonatal intensive care unit and to treat critically ill infants who experience severe anemia due to physiologic and iatrogenic factors [1–4]. Nearly 80% of all infants born at <27 weeks of gestational age receive one or more RBC transfusions during their birth hospitalization [5–8]. Though repletion with packed RBC has obvious benefits (such as increased oxygen-carrying capacity), transfusions have also been implicated in the subsequent development of necrotizing enterocolitis (NEC) within 48–72 h after transfusion [7–15]. NEC is the most common acquired gastrointestinal disease of premature neonates, affecting 5%–15% of infants born with <1,500 g [5–8,16–18] and, despite technological advancement, remains a leading cause of death in neonates born between 22 and 28 weeks gestation [9–12,19,20]. NEC is also associated with multiple immediate serious complications, such as death due to sepsis, and long-term complications, including intestinal failure, growth delay, and adverse neurodevelopmental outcomes [21,22]. The causes of NEC are diverse; however, our recent findings documented that phlebotomy-induced anemia resulted in a disproportionate and persistent increase in intestinal permeability in pre-weaned mice because of the disruption of epithelial adherens junctions [23], developing a low-grade inflammatory state in the intestine with prominent macrophage precursor infiltration. Subsequent RBC transfusions activate these macrophages and thus cause NEC-like injury [24]. To examine the pathogenesis of NEC, various murine models have been established. This study will describe an improvement upon the previous version and a detailed protocol of our murine model of RBC transfusion–associated NEC.

## Materials and reagents

**Biological materials**

1. *Serratia marcescens* (American Type Culture Collection, catalog number: 13880)
2. C57BL/6J mice (The Jackson Laboratory, catalog number: 000664)

**Reagents**

1. Citrate phosphate dextrose adenine (CPDA-1) (Sigma, catalog number: C4431)
2. Luria-Bertani (LB) broth (Quality Biological, catalog number: 340004101)
3. Normal saline (0.9% sodium chloride) (Sigma-Aldrich, catalog number: 7647145)
4. Trypan blue (Thermo Fisher, catalog number: 15250061)
5. 0.5% Proparacaine hydrochloride ophthalmic solution (Alcon Laboratories, Inc., catalog number: NDC0998-0016-15)
6. Phosphate-buffered saline (PBS) (Gibco, catalog number: 10010023)
7. Intestinal fatty acid binding protein ELISA kit (MyBioSource, catalog number: MBS035456)

**Laboratory supplies**

1. Cell pack (Sysmex America, model: DCL 300A)
2. Sterile Acrodisc WBC syringe filter (Cytiva, catalog number: AP-4952)
3. Magnifier lens
4. 1.5 mL Eppendorf tubes (Eppendorf, catalog number: 022363204)
5. 14 mL culture tube (Corning, catalog number: 352057)
6. 1 mL syringe (Thermo Fisher, catalog number: 309625)
7. 0.3 mL insulin syringe (Becton, Dickinson and Co., model: BD Ultra-Fine II)
8. Umbili-cath polyurethane UVC catheter, single lumen, 3.5 French (Utah Medical, catalog number: 4183505)

## Equipment

1. Weighing balance (Mettler-Toledo, catalog number: ME104TE)
2. Sysmex XN-1000TM hematology analyzer (Sysmex Corp, IL)
3. Spectrophotometer (Molecular Devices, model: SpectraMax Plus)

## Procedure

- **Preparing packed stored RBCs for transfusion**
  Time duration: 30 min
  1. One week before transfusion, anesthetize FVB/NJ adult donor mouse with isoflurane (1.5%–2.5%) in an induction box until the mouse is non-responsive and apply 0.5% proparacaine ophthalmic solution 5 min before whole blood retro-orbital bleeding.
  2. Prepare a 1.5 sterile microliter centrifugal tube with a final concentration of 14% (140 μL) of CPDA-1 solution and 860 μL of whole blood collected from each adult mouse to make a combined volume of 1 mL.
  3. Standard heparinized or nonheparinized microhematocrit capillary tubes can be used. Hold the animal by the back of the neck and tighten the loose skin of the head with the thumb and middle finger to keep the animal stable. Place the tip of the capillary tube at the medial canthus of the eye under the nictitating membrane.
  4. With a gentle thrust and rotation motion past the eyeball, the tube will enter the slightly resistant sinus membrane. The eyeball itself remains uninjured. As soon as the sinus is punctured, blood enters the tubing by capillary action. When the desired amount of blood is collected, withdraw the tube and apply slight pressure to the eye with a clean gauze pad to ensure hemostasis. Take care not to scratch the cornea with the gauze pad.
  5. Euthanize the mice in accordance with IACUC and institutional policies.
  6. Immediately after collection, leukoreduce the whole blood using a sterile Acrodisc WBC syringe filter. Then, centrifuge the leukoreduced blood at 295× g for 10 min and partially remove the supernatant to obtain a hematocrit of approximately 75%. Transfer the blood to 1.5 mL centrifugal tubes to create multiple aliquots of 500 μL, leaving a small residual air space, and store in the dark at 4 °C until use.
- **Preparing *Serratia marcescens***
  Considering the potential importance of enteric Gammaproteobacteria in NEC pathogenesis, we introduced a well-characterized strain of *Serratia marcescens* in our mice on postnatal day 7 (P7) to achieve fecal Gammaproteobacteria abundance similar to what is seen in premature infants. *Serratia sp.* has been previously used as prototypical Gram-negative bacteria in rodent models of NEC based on several characteristics: (a) translational relevance, as they were originally isolated from a premature infant with NEC; (b) non-pathogenicity in mice, as mice colonized with these bacteria in our laboratory have remained asymptomatic for several months with normal body growth and no histological evidence of intestinal inflammation; and (c) natural red pigmentation of *Serratia* colonies, which facilitates detection in fecal/tissue cultures.
  Time duration: 10 min
  1. Three days before transfusion, use a sterile pipette tip to scrape approximately 10 μL of an enteric bacterial glycerol stock from a frozen cryovial.
  2. Place the sterile pipette tip containing the bacterial aliquot into a 15 mL culture tube containing 10 mL of LB broth. Culture the bacteria overnight (16 h) in an orbital shaker at 37 °C with agitation speed at 150 rpm. A control culture tube with 10 mL of sterile LB broth should also be cultured simultaneously in the orbital shaker to ensure there is no concern for bacterial contamination of the LB broth.
  3. Use a spectrophotometer to measure the culture density at 600 nm (OD_600nm_). Add 1 mL of sterile LB broth into a 1 cm cuvette and measure the OD_600nm_ to serve as the blank. In a separate 1 cm cuvette, add 1 mL of the *S. marcescens* culture and measure the OD_600nm_.
*Note: The OD_600nm_ value should be 0.6 ± 0.02, corresponding with the exponential bacterial growth phase. If the OD_600nm_ is greater than 0.6, use LB broth to dilute the culture until the diluted culture exhibits the targeted OD_600nm _value. If the OD_600nm_ is less than 0.6, continue to culture the inoculum until the OD_600nm_ reaches 0.6.*
  4. Once the *S. marcescens* culture has achieved the targeted OD_600nm_, transfer 2 mL of the culture to 2 mL centrifuge tubes and centrifuge at 3,000× *g* for 10 min. Discard the supernatant.
  5. Resuspend the bacterial pellets each in 1 mL sterile PBS for oral gavage to mouse pups.
- **Phlebotomy-induced anemia**
  We have improved our previously published protocol by retrieving a smaller blood volume (10 μL per gram of body weight) daily rather than 20 μL on alternate days. This change reduced animal stress and anxiety and prevented acute losses of plasma volume while continuing to achieve effective hematocrits of 18%–23%.
  Time duration: 10 days
  1. On the first day of the experiment, in the animal facility, weigh the P1 mouse pups and randomly assign the animal into one of two experimental groups: anemic or control groups.
    - Anemic pups: Gently hold the mouse pups by grasping the loose skin at the base of the neck and perform facial vein phlebotomy to remove 10 μL of blood per gram of body weight **(Figure 1)**.
*Note: Restrain the mouse gently. Figure 1 shows a detailed enlarged representation of the approximate area of the facial vein by measuring the length of the eye below the lateral canthus and the width of the eye caudally.*
      **Figure 1.**
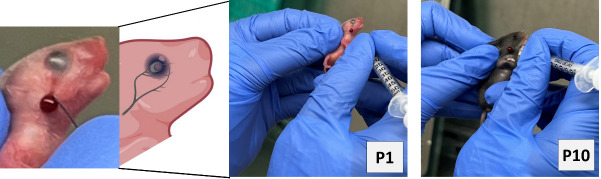
Facial-vein phlebotomy in postnatal day 1 (P1) and P10. Hematocrit levels will be measured in blood specimens using Sysmex XN-1000TM hematology analyzer according to their standard procedures. To maintain plasma volume, an aliquot of normal saline (0.9% sodium chloride) equal to the amount of blood removed was administered intraperitoneally by carefully inserting a 30 G needle fixed to a 0.5 mL syringe.
    - Control groups: Prick the control pups with a needle through the scruff of the neck while ensuring that they do not bleed in order to subject all mice to similar handling and stress.
*Note: The facial vein in murine neonates is well visible during P1–4 days; after P5 fur has developed, which reduces the visibility of the facial vein. Even so, facial vein phlebotomy in P5–10 animals is possible by keeping the mouse pups under warm lights for 5 min to dilate the veins for phlebotomy.*
  2. Repeat the above steps for both anemic and control group animals every day until P10. In addition to measuring the hematocrit during each phlebotomy, thus confirming the level of anemic condition, visually monitor the anemic mouse pups in the anemic group for the gradual development of pallor. Figure 2 shows the anemic mice visibly pale in the face and toes compared with the control mice following a reduction in hematocrit.
*Note: The size and weight of each experimental mouse pup are monitored and recorded during phlebotomy and after RBC transfusion.*
    **Figure 2.**
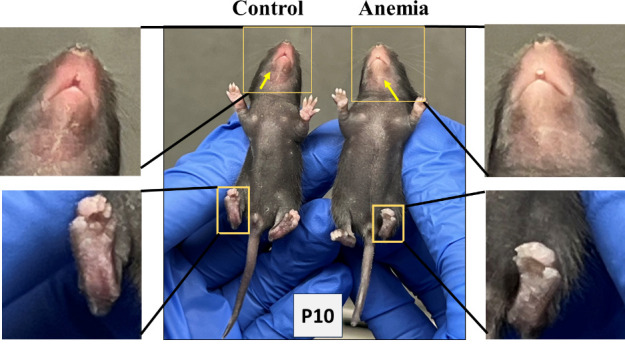
Visible pallor in anemic mice.
  3. On P7, administer oral gavage feedings of *S. marcescens* to the mouse pups of both the control and anemic groups.
    - Fill a 1 mL syringe with *S. marcescens* culture suspension and attach it to a 3.5 French umbili-cath polyurethane UVC catheter.
    - Gently hold the pup by grasping the loose skin at the base of the neck and use forceps to grasp the distal end of the catheter. Gently introduce 2 cm of the catheter into the oropharynx and esophagus (Figure 3). There should be no significant resistance with the insertion of the catheter.
    - Slowly dispense 50 μL (10^4^ CFU) into the stomach.
    - Slowly withdraw the catheter from the oral cavity.
    - Monitor the animal for increased respiratory effort or emesis associated with a mispositioned catheter.
    **Figure 3.**
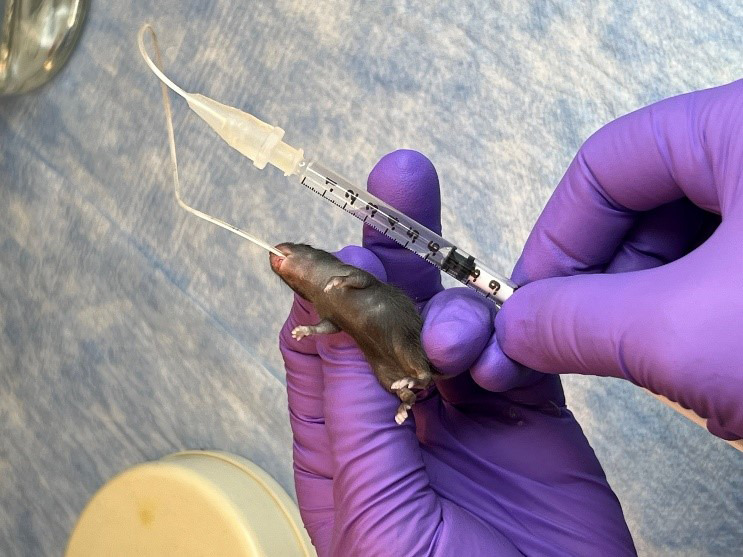
Oral gavage of *Serratia marcescens* using 3.5 French catheter.
- **Transfusion of stored RBC transfusion by orbital-sinus injection**
  On P11, randomly assign a few mouse pups from the above anemic and control groups again into two experimental groups: anemia–RBC transfusion and control–RBC transfusion groups. Both group animals are transfused as below:
  1. Bring the stored RBCs from the refrigerator and gently resuspend them by rotating tubes in the rotator at room temperature.
  2. To administer retro-orbital injections in pups, use a 31 G, 0.3125 in needle attached to a 0.3 mL insulin syringe. Do not inject more than 50 μL of liquid in each orbital sinus. The pups are not anesthetized for this procedure, because they can be adequately manually restrained without being anesthetized.
  3. For neonatal mice, right-handed lab personnel will find it easiest to place the pup in left lateral recumbency, with their head facing right, and administer the injection into the right retro-orbital sinus. This procedure can be reversed to accommodate left-handed personnel to inject into the left side of retro-orbital sinus (Figure 4).
    **Figure 4.**
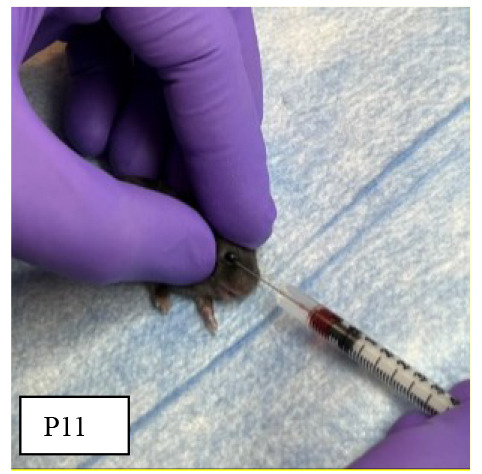
Orbital sinus injection of stored red blood cells (RBCs)
  4. To test the efficiency of orbital sinus injection, extra mouse pups may be injected with Trypan blue and monitored for body color change to confirm systemic circulation (Figure 5).
    **Figure 5.**
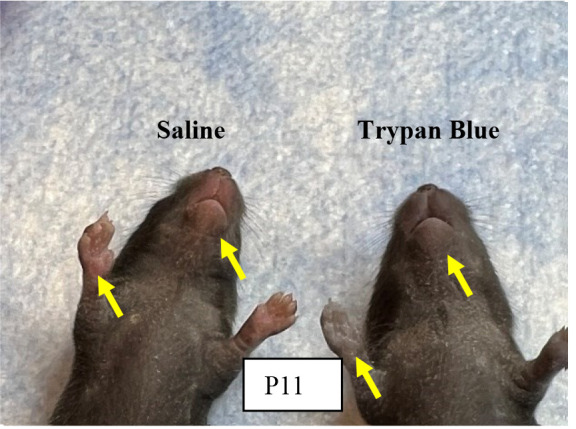
Effectiveness of orbital sinus injection evaluated with Trypan blue.
*Note: Tryan blue injection helps to confirm the efficiency of our intravenous injection method via orbital sinus in neonatal mice and it does not vary in either experimental group.*
  5. Gently restrain the pup’s head with the tip of the thumb and forefinger. Lab personnel must be careful not to place pressure on the trachea or impede venous flow. Nestle the rest of the pup’s body between the thumb and forefinger. In our experience, once the mouse is comfortably restrained, there is minimal struggle, and the mouse does not emit audible vocalizations.
*Note: Use sterile saline and a cotton-tipped applicator to gently clean the area above the eye. This helps to remove any skin flakes that may get in the way of the injection and helps to make the skin slightly more transparent.*
  6. Care must be taken not to overly wet the pup because this could increase the risk of hypothermia. We do not use alcohol or a topical ophthalmic anesthetic. The ophthalmic anesthetic will not penetrate the skin, and we think that alcohol might irritate the pup’s facial skin.
  7. Insert the needle, bevel down, at the 3 o’clock position into the eye socket (the area that will become the medial canthus) at an angle of approximately 30°. Mentally visualize the back of the socket and advance the needle to the area of the retro-orbital sinus.
  8. Make the injection in a gentle, smooth, fluid motion. If the injection is successful, the lab personnel might observe blanching of the superficial temporal vein, but this does not always occur. Regardless of whether blanching is noted, we have seen the injectate in the target organs.
  9. Withdraw the needle slowly, allowing the injectate to redistribute. We sometimes see a small drop of blood at the injection site, which can be gently cleaned with a cotton-tipped applicator.
  10. Place the pup in the second prepared nest. When all the pups in a group have received injections, check each one for any additional bleeding and clean the blood, if necessary. Return the pups to their mother in the home cage.
- **Development of NEC after RBC transfusion**
  1. Check hematocrit values immediately after each phlebotomy and after RBC transfusion. Figure 6 shows a steady reduction from 51% ± 1.54 to 22.5% ± 0.67 from P1 to P11 in anemic mice groups. After RBC transfusion on P11 in a typical experiment, the hematocrit increased to 40.33% ± 1.28 in anemic groups versus 58.67% ± 1.11 in control groups.
  2. After the RBC transfusion (on P11), sacrifice pups on P12 according to the institutional ethical guidelines, collecting blood and intestinal tissues for further analyses.
    **Figure 6.**
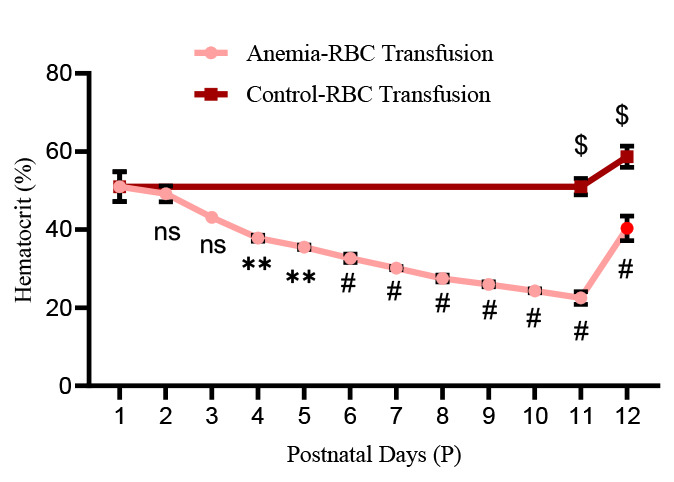
Hematocrit values in experimental groups: Line chart (mean ± SE) demonstrating serial reduction of hematocrit (Hct in %) during the sequence of daily phlebotomy in P1–10 mouse pups and a significant increase in hematocrit after red blood cell (RBC) transfusion in both anemic and control mouse pups. N = 6 mice per group. *p < 0.05; Šídák's multiple comparisons test. **p < 0.01, *#p <* 0.001 vs. P1 baseline; ^$^ *p <* 0.001 vs. pre-transfused.
  3. Intestinal injury is marked by measuring intestinal fatty acid binding protein (i-FABP2) concentrations in the plasma of all four groups using a commercially available ELISA kit per the manufacturer’s protocol. The assay has a linear range of 78–5,000 pg/mL. As depicted in Figure 7, i-FABP2 levels were significantly increased in anemic-transfused groups compared with others.
    **Figure 7.**
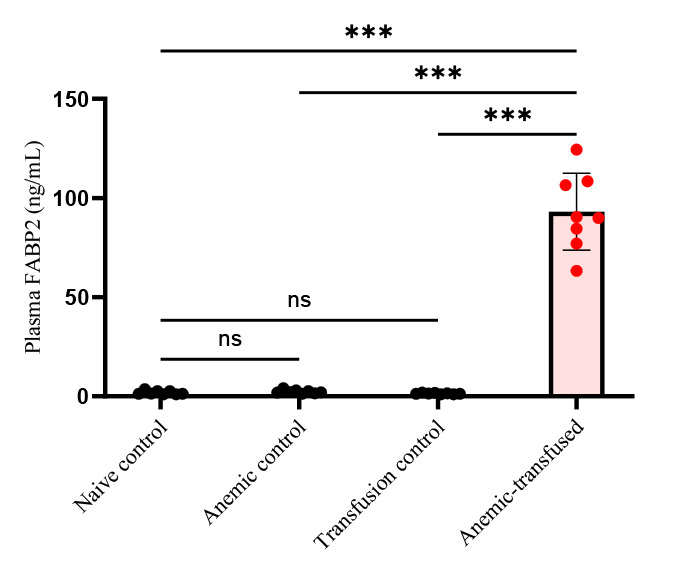
Intestinal injury marker of plasma fatty acid binding protein (FABP2) level in experimental groups. Bar diagram (mean ± SE) summarizes plasma intestinal fatty acid-binding protein 2 (iFABP2) concentrations in naïve control, anemic control, transfusion control, and anemic-transfused mice. N = 8 mice per group. ***p < 0.001; Šídák's multiple comparisons test.
  4. Consistent with FABP2 levels, histopathology analyses show that anemic mouse pups that received RBC transfusion developed intestinal injury in the ileocecal region with complete disruption of the crypt-villus axis, severe separation of the lamina propria and transmural necrosis (Figure 8).
    **Figure 8.**
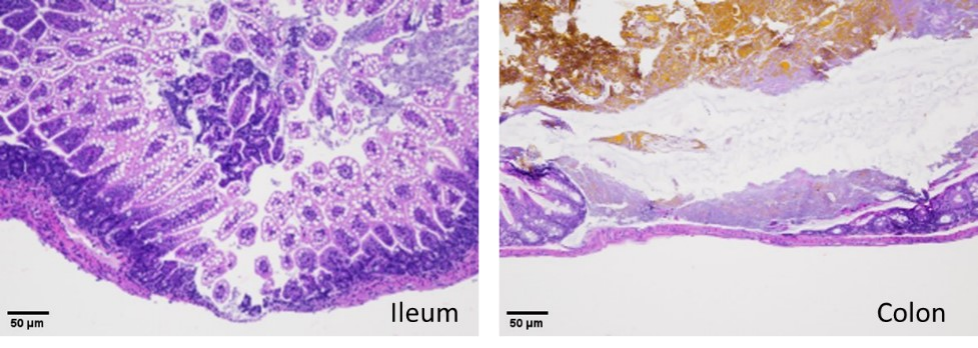
Hematoxylin–eosin staining of the ileum (left) and colon (right) shows necrotizing enterocolitis (NEC) injury in anemic-transfused mice.

## Validation of protocol

This protocol or parts of it has been used and validated in the following research article(s):

- MohanKumar, K. et al. (2019). A murine neonatal model of necrotizing enterocolitis caused by anemia and red blood cell transfusions. Nature Communication.

## General notes and troubleshooting

**Troubleshooting**

**Problem 1**

The experimental model solely relies on neonates; therefore, handling plays a crucial role.

**Potential solution**

Pups need to be held in appropriate positions by giving them maximum comfort. Make sure to reduce the stress while performing phlebotomy to the maximum. Do not disturb the nest while taking the pups away from the cage. Use soft facial tissues to wipe off the blood during phlebotomy. Wait until the dam moves away and gives some space to handle the pups. Making sure to clean the blood from the pups after bleeding improves their survival, because if the dam senses the blood smell, it may ignore the pups while nursing.

**Problem 2**

Identification of facial veins in neonatal mouse pups for phlebotomy.

**Potential solution**

The facial vein in murine neonates is well visible during P1–4 days; then, once fur has developed, it reduces the visibility of the facial vein. P5–10 mouse pups are kept in a box under warm lights to dilate the veins, which helps to visualize the dilated “skin dune” for phlebotomy.

**Problem 3**

Pups develop respiratory distress during oral gavage of *S. marcescens* using a 3.5 French catheter.

**Potential solution**

If the catheter is inserted into the trachea, it should be immediately withdrawn and reinserted back slowly. Ensure that there is no significant resistance to the insertion of the catheter.

**Problem 4**

Administration of stored RBCs may cause circulatory overload.

**Potential solution**

Acute administration of stored RBCs to neonatal mouse pups may cause circulatory overload, characterized by acute respiratory distress, tachycardia, increased blood pressure, and acute pulmonary edema. To reduce the risk of these iatrogenic conditions, it is important to carefully monitor the neonatal mouse pups after injection and maintain a warm temperature using warm lights. It is also important to train personnel on careful handling and intravenous injection of pups to avoid unintentional death. During the procedure of phlebotomy and/or RBC transfusion, if any of the experimental mouse pups display signs of distress, pain, or discomfort, they should be euthanized and autopsied to determine the cause of death.
